# Tuning Pore Sizes of Core–Shell Dendritic Mesoporous Silica Nanoparticles for Efficient Loading of Functional Materials

**DOI:** 10.1021/acs.langmuir.6c02682

**Published:** 2026-07-04

**Authors:** Olapeju G. Oyedepo, Kevin Wittchen, Kurosch Rezwan, Sascha Beutel, Michael Maas

**Affiliations:** † Advanced Ceramics, 9168 University of Bremen, Am Biologischen Garten 2, 28359 Bremen, Germany; ‡ Institute of Technical Chemistry, Leibniz University Hannover, Callinstraße 3-5, 30167 Hannover, Germany; § MAPEX Center for Materials and Processes, University of Bremen, Bibliothekstraße 1, 28359 Bremen, Germany

## Abstract

Precise control over pore accessibility in dendritic mesoporous silica nanoparticles (DMSNs) is essential for loading and transport of large biomolecules and functional nanoparticles for catalysis, sensing, and further applications. Here, we present a scalable anion-assisted synthesis using sodium salicylate to tune pore size and shell thickness in nano- (170 nm) and microscale (1 μm) DMSNs core–shell particles. By varying the core-to-silane ratio, complex pore structure can be adjusted without altering the surfactant composition. Increasing the relative core content reduces silane availability per particle and suppresses secondary micelle filling, eliminating small mesopores (∼4 nm) while preserving large radially oriented interwrinkle mesopores (12–21 nm), consistent with a micelle-filling growth mechanism. This approach yields shell thicknesses ranging from ∼19 ± 5 nm to 87 ± 7 nm for small cores and from ∼11 ± 17 nm to 151 ± 23 nm for large cores. Loading experiments with silver nanoparticles (∼4 nm) and lysozyme (∼4 nm) reveal that diffusion limitations, rather than total surface area, control uptake in these materials. Core–shell particles dominated by large, accessible interwrinkle mesopores exhibit substantially higher loading than structures containing a higher fraction of small mesopores. Surface-area-normalized lysozyme loading reaches 4.1 ± 1.1 mg m^–2^ for large-core (1 μm) particles with predominantly open interwrinkle channels, demonstrating that small mesopores can significantly reduce effective accessibility despite increasing nominal surface area. These results establish the core-to-silane ratio as a simple and robust parameter for engineering hierarchical pore architectures in DMSNs and provide a practical framework for designing mesoporous hosts for enzymes, nanocatalysts, and other nanoscale guests.

## Graphical abstract

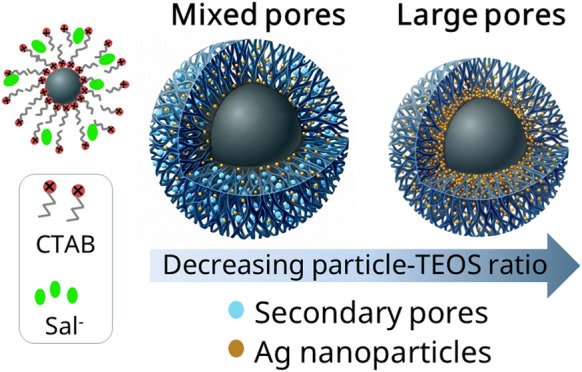

## Introduction

Dendritic mesoporous silica nanoparticles (DMSNs), also known as dendritic fibrous nanosilica (DFNS), wrinkled mesoporous silica (WMS), or KCC-1 (KAUST Catalysis Centre),^1,2^ exhibit a unique wrinkled lamellar morphology in which radially oriented pore channels extend from a dense core, forming a fibrous (or “wrinkled”) structure with high surface area, large pore volume, and tunable pore diameters typically ranging from 3 to 25 nm.^2–7^ However, standalone mesoporous nanoparticles often lack sufficient functionality, structural robustness, and accessibility for practical flow-through or separation-based applications. Core–shell architectures incorporating mesoporous silica shells address these limitations by combining the inherent properties of solid cores with the tunable porosity and high surface area of mesoporous silica. Since pore size dictates molecular accessibility within mesoporous networks, precise control of pore size and pore size distribution of silica particles with a lamellar shell is essential for optimizing performance in enzyme immobilization, catalysis, separation, cell lysis, and other biotechnology applications.^8–10^ Therefore, developing simple and scalable synthesis strategies for engineering the pore size of core-lamellar shell particles remains critical for expanding the practical use of mesoporous silica-based materials.

The synthesis of DMSN nanosilica can be broadly classified into two main approaches: the microemulsion method and the anion-assisted synthesis method. The microemulsion method uses an oil phase (*e.g*., cyclohexane,^1,2^ octane,^11^ ethyl ether,^12^ or hydrophobic organosilanes^13–15^ ), an aqueous phase, cetyltrimethylammonium bromide (CTAB) as surfactant, and a cosurfactant to form bicontinuous droplets that template DMSNs. Pore size control is achieved by adjusting lamellar density, often via primary alcohol cosurfactants of varying chain lengths.^16^ For core–shell architectures, 1,3,5-triisopropylbenzene (TIPB), a swelling agent, can expand pores, producing Fe_3_O_4_@DMSNs-OTES with reported pore sizes of 10–60 nm, although most pores were 5–10 nm.^17^ The method has also been applied to DMSN shells on microsphere silica cores, where stirring rate and conditions tune shell thickness (13–200 nm) and pore size (5–28 nm) in a single-pot synthesis.^10,18^ These monodispersed core–shell particles maintain high surface area and demonstrate good chromatographic performance, combining high separation efficiency with low back pressure. However, reproducible synthesis with the microemulsion approach is challenging because the reaction is highly sensitive to small changes in the complex formulation, mixing kinetics, and overall volumes, and the method requires large amounts of organic solvents, which limits the sustainability and scalability of DMSN production.

In contrast, the anion-assisted synthesis method, also known as the dual templating approach, is an aqueous, environmentally friendly method for DMSN synthesis that relies on the use of cationic surfactants with larger counter-anions such as cetyltrimethylammonium tosylate,^19^ or a combination of conventional cationic surfactants such as CTAB with anionic or nonionic surfactants such as SDS^20^ or small organic anions like sodium salicylate.^21,22^ The overall dendritic pore size and architecture are strongly influenced by the choice of cosurfactant,^19,20,23^ cosurfactant/surfactant ratio, reaction temperature, precursor concentration, and counterion hydrophobicity.^24^

Despite extensive investigation, the mechanism of DMSN formation and the evolution of pores in anion-assisted aqueous systems remains unclear. The key question is how anions influence micelle–silicate assembly and the evolution of the characteristic center-radial pore structure. In the postulated “weak templating” mechanism, counterions compete with silicate oligomers for association with cationic micelles, generating partially silica-coated micelles.^19,20,25,26^ These assemble into blocks that aggregate into dendritic frameworks. Large pores arise from interblock spacing, while smaller mesopores are templated by individual micelles. Direct evidence for partially coated micelles is limited, and the model does not fully explain uniform silica walls.^27^ Alternatively, the micelle-filling mechanism proposes that anions penetrate the micelle interior, similar to microemulsion-like behavior.^21,24,28^ In this view, anion insertion alters the surfactant packing parameter, driving micellar structural transitions (*e.g*., cylindrical or wormlike to vesicular or lamellar phases) that ultimately serve as templates or building blocks for dendritic or lamellar pores. Furthermore, silica-coated micelles heterogeneously nucleate on preformed large lamellar pore surfaces, leading to intermediate structures with both large and small mesopores originating from interparticle packing voids and eventually forming pomegranate-like MSNs,^24^ where the dendritic pores are filled. Both mechanisms share a critical feature: the dendritic structure is not static but dynamically evolves during condensation.

Beyond their formation, the complex pore architecture of DMSNs strongly influences the transport of large guest molecules. While all mesopores contribute to surface functionality, diffusion of bulky molecules is primarily controlled by the pore size and connectivity. Large radial channels enable rapid transport into the particle interior, whereas smaller mesopores embedded within these channels can create diffusion bottlenecks, slowing molecular movement, despite providing additional surface area.^29^ This generates a trade-off between diffusion efficiency and surface functionality, as molecules must navigate narrower pores to access the full internal surface.^30,31^ Designing lamellar dendritic silica with appropriately sized, interconnected pores is therefore essential to maximize both molecular transport for large species and available functional surface area.^32,33^

With this background, we explore an anion-assisted synthesis approach to fabricate silica core–shell particles with tailored dendritic fibrous nanosilica (DFNS) coatings. Sodium salicylate (NaSal), a known structure-directing agent for DFNS formation,^21^ is employed here to mediate the growth of DFNS-type mesoporous shells on preformed silica cores. The novelty of this work lies in extending this anion-assisted DFNS coating strategy to silica cores of different sizes and systematically correlating the core-to-TEOS mass ratio with the resulting shell thickness and mesostructural properties. This approach is demonstrated on representative silica cores at both the nanoscale (170 nm) and microscale (1 μm), enabling controlled synthesis of hierarchical core–shell architectures. Nanoparticles in the ∼170 nm range, such as magnetic^34,35^ or zeolitic cores,^36,37^ are commonly employed in applications requiring facile separation or catalytic functionality. Micrometer-sized silica cores are typically used in chromatographic separations,^10,18^ where excessive packing of smaller particles would generate high back pressure, while larger particles provide improved flow-through characteristics in columns.

We further study the accessibility of the pore structures by loading them with lysozyme and positively charged silver nanoparticles, which are used as proxies for functional biomolecules and nanoparticles, *e*.*g*., with antibacterial, catalytic, or plasmonic applications. Larger pore core–shell particles are expected to provide enhanced accessibility and higher loading efficiency due to unobstructed substrate diffusion.

## Materials and Methods

### Chemicals

The 170 nm Stöber silica (SiO2P015-01) and 1 μm silica (SiO2P100-01) were purchased from Fiber Optic Centre Inc. Triethanolamine (TEA, ≥99.0%, 90279), sodium salicylate (NaSal, ≥99.5%, 71945), tetraethyl orthosilicate (TEOS, >99%), polyethylenimin (PEI, 25 kDa, 408727), lysozyme (from chicken egg white, L6876), and phosphate-buffered saline (PBS, P4417) were purchased from Sigma-Aldrich Chemie GmbH, Munich, Germany. Cetyltrimethylammonium bromide (CTAB, 98%, A15235.36) was purchased from Thermo Scientific.

### DMSNs and Core–Shell DMSNs Synthesis

DMSN particles were synthesized based on Yang et al.^21^ A solution of 0.068 g triethanolamine (TEA) in 25 mL deionized water was stirred at 80 °C in an oil bath under magnetic stirring for 30 min. Afterward, 380 mg of cetyltrimethylammonium bromide (CTAB) and 168 mg of sodium salicylate (NaSal) were added (molar ratio NaSal/CTAB = 1), and stirring was continued for 1 h. Next, 4 mL of tetraethyl orthosilicate (TEOS) was added dropwise to the solution under gentle stirring, and the reaction mixture was stirred for an additional 2 h. The resulting product was collected by high-speed centrifugation at 5000 rpm for 15 min (Heraeus Megafuge 16, Thermo Fischer Scientific), washed several times with ethanol to remove residual reactants, and dried overnight at 80 °C. The final material was then calcined at 550 °C for 6 h to remove the surfactant template.

To coat DMSNs onto silica core particles, a given mass of core particles was dispersed in 10 mL of deionized water and sonicated for 30 min to ensure uniform dispersion. Separately, 0.068 g of triethanolamine (TEA) was dissolved in 15 mL of deionized water and stirred at 80 °C in an oil bath under magnetic stirring for 30 min. The core particle suspension was then added and stirred for an additional 10 min. The standard anion-assisted synthesis of DMSNs was subsequently carried out without modification, using the same reagent volumes for all samples. Core–shell particles with small cores (170 nm) are referred to as DMSNs@Ssilica-R, while those with large cores (1 μm) are referred to as DMSNs@Bsilica-R, where *R* denotes the mass ratio of core particles to TEOS. The core-to-TEOS ratio was carefully controlled to favor heterogeneous shell growth on preformed cores, and under these conditions, no secondary nucleation of core-free DMSN particles was detected.

### Material Characterization

The ζ-potential of the nanoparticles was measured using a Zetasizer Nano SP (Malvern Instruments Ltd., Worcestershire, UK). For the transmission electron microscope (TEM) analysis, 1 wt % particle suspension was prepared and sonicated for 15 min. Thereafter, 40 μL of the suspension was diluted with ethanol and sonicated for 5 min to ensure proper dispersion of the particles. Of this diluted suspension, 2 μL were dropped on a TEM grid (Formvar, 3.05 mm copper 200 mesh, Plano GmbH, Germany). The samples were dried in an evacuated desiccator for at least 2 days. Micrographs of the particles were taken with a TEM (Zeiss 900 A, Carl Zeiss, Germany) with an acceleration voltage of 80 kV. The equivalent diameter of individual particles was determined from area measurements using ImageSP (TRS, Germany) software. Shell thickness was calculated as the difference between the average diameter of the coated particle and the average diameter of the core particle, divided by two to account for diameter-to-radius conversion, with the associated uncertainty obtained by propagating the standard deviations of both measurements. STEM-EDS elemental maps were acquired using a Thermo Fisher Scientific Spectra 300 Scanning Transmission Electron Microscope.

SEM micrographs of the synthesized particles were obtained with a scanning electron microscope (SEM, Supra 40, Carl Zeiss, Germany) with an acceleration voltage of 15 kV. Materials were fixed on an SEM sample stub by using adhesive carbon pads.

Nitrogen adsorption–desorption measurement was conducted by a Belsorp-Max, Bel Japan Inc. system at −196 °C after drying the samples at 120 °C at reduced pressure (<2 mbar). The total pore volume was calculated based on the adsorbed amount at the maximum relative pressure (*P*/*P*_0_) of 0.99. The pore size of samples was calculated through the Barrett–Joyner–Halenda (BJH) method from the desorption branches of the isotherms. The Brunauer–Emmett–Teller (BET) method was used to calculate the specific surface areas. Two independent measurements were performed for each sample, and average values are reported. The dominant experimental uncertainty arises from the determination of the sample mass during weighing (balance readability ± 0.1 mg).

### Synthesis and Analysis of Ag-PEI Impregnated DMSNs Core–Shell Particles

PEI-capped silver nanoparticles (Ag-PEI) were synthesized in the dark by mixing 36.5 mL DI water, 10.0 mL AgNO_3_ (10 mM), and 23.5 mL PEI (20 mg mL^–1^) for 10 min, followed by rapid addition of 30.0 mL ice-cold NaBH_4_ (10 mM). The mixture was stirred in an ice bath for 30 min, yielding nanoparticles with a ζ-potential of +16.8 ± 1.0 mV. Ag-PEI particles were impregnated into DMSNs core–shell materials with a ζ-potential value of −28.0 ± 1.0 mV by sonication (5 min) and incubation (90 min), and then deposited on TEM grids and vacuum-dried. RGB representations of STEM-EDS Si–Ag maps were analyzed in Python: channels were separated, the Si map defined the particle center and segmented core and particle boundaries, and the shell was divided into concentric 10 nm layers. After background correction, Ag intensity was quantified per shell and reported as both total intensity and percentage of total Ag versus distance from the surface of the 2d projection of the core.

### Enzyme Immobilization on Core–Shell DMSNs

DMSNs or core–shell DMSN particles (2 mg) were dispersed in 1 mL of PBS and mixed together with 1 mL of lysozyme solution (2 mg mL^–1^ in PBS solution) in 2 mL centrifuge tubes. Then, the mixture solution was incubated for 24 h at room temperature. The lysozyme-loaded particles were collected after centrifugation (15 000 rpm, 10 min). The collected supernatant was measured by a UV–vis spectrophotometer at 280 nm to measure the amount of lysozyme loaded in the particles. The residual lysozyme content was calculated by the difference in the lysozyme concentration before and after loading. All of the experiments were performed in triplicate.

## Result and Discussion

DMSNs were synthesized via an anion-assisted method in an alkaline aqueous system using tetraethyl orthosilicate (TEOS) as the silica precursor, cetyltrimethylammonium bromide (CTAB) as the surfactant, and sodium salicylate as a co-template in a 1:1 molar ratio with CTAB.

TEM and SEM images revealed the typical wrinkled lamellar structure of DMSNs (Figure 1a,b). BJH pore size analysis (Figure 1c) reveals a wide pore distribution that starts with a maximum at ∼2 nm and then gradually falls off to 28 nm, with the majority of pores concentrated between the beginning of the detectable mesoporous range and about 22 nm corresponding to radially oriented channels. This contrasts with Yang et al.,^21^ who observed a single peak at 17.5 nm for the same CTAB-sodium salicylate ratio, but this is consistent with other studies where low catalyst (triethanoamine)-to-TEOS ratios and extended reaction times yield bimodal pore distributions.^17,23^

**Figure 1:**
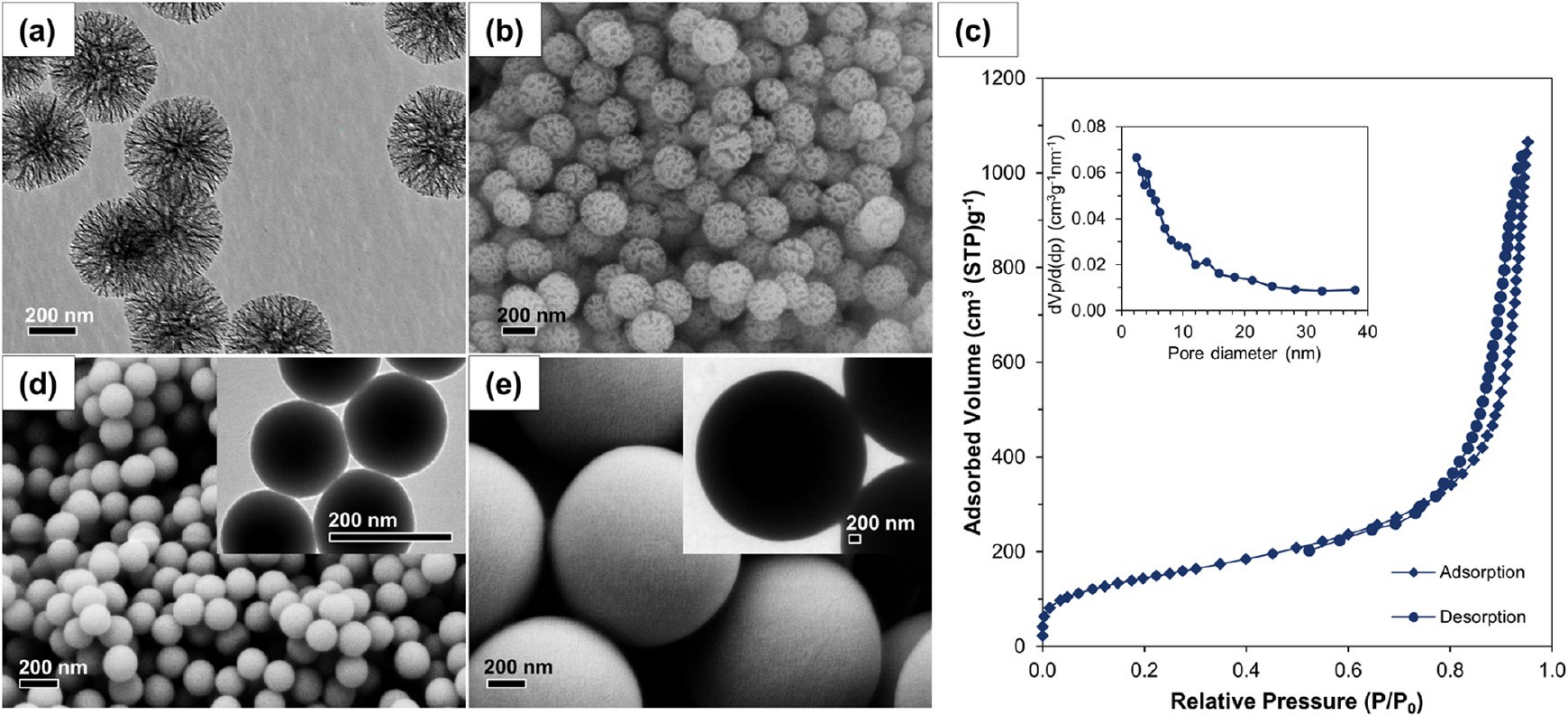
(a) TEM image, (b) SEM image, and (c) nitrogen adsorption–desorption isotherm with corresponding pore size distribution of DMSNs synthesized via the anion-assisted method. The DMSNs (*d* ≈ 210 nm) exhibit a BET surface area of 436 m^2^/g and a pore volume of 1.64 cm^3^/g. SEM images with TEM insets of (d) small and (e) large silica cores before DMSNs coating.

The effect of increasing core particle mass on the morphology and pore distribution of DMSNs@silica was investigated using 170 nm and 1 μm Stöber silica spheres. SEM and TEM images of core particles (Figure 1d,e) show a smooth and featureless surface. After coating with DMSNs shell using the anion-assisted synthesis method, TEM images (Figure 2a–d) show that DMSNs@Ssilica and DMSNs@Bsilica exhibit the characteristic center-radial wrinkled structure of DMSNs, consistent with previous reports.^27,38^ TEM images of DMSNs@Ssilica and DMSNs@Bsilica (Figure 3a–d) also show a dense core structure, which was not observed in discrete DMSNs, signifying the successful coating of the cores with the DMSNs shell. A well-defined and uniform core–shell morphology with decreasing shell thickness from 87 ± 7 nm to 30 ± 6 nm is observed up to *R* = 4 for DMSNs@Ssilica, where *R* denotes the mass ratio of core particles to TEOS used during the shell-growth process. However, DMSNs@Ssilica-2 shows incomplete and nonuniform shell coverage, indicating disruption of controlled shell growth. The average particle diameters measured from TEM images are 346 ± 11, 254 ± 8, 232 ± 8, and 211 ± 8 nm for DMSNs@Ssilica-18, DMSNs@Ssilica-7, DMSNs@Ssilica-4, and DMSNs@Ssilica-2, respectively.

**Figure 2:**
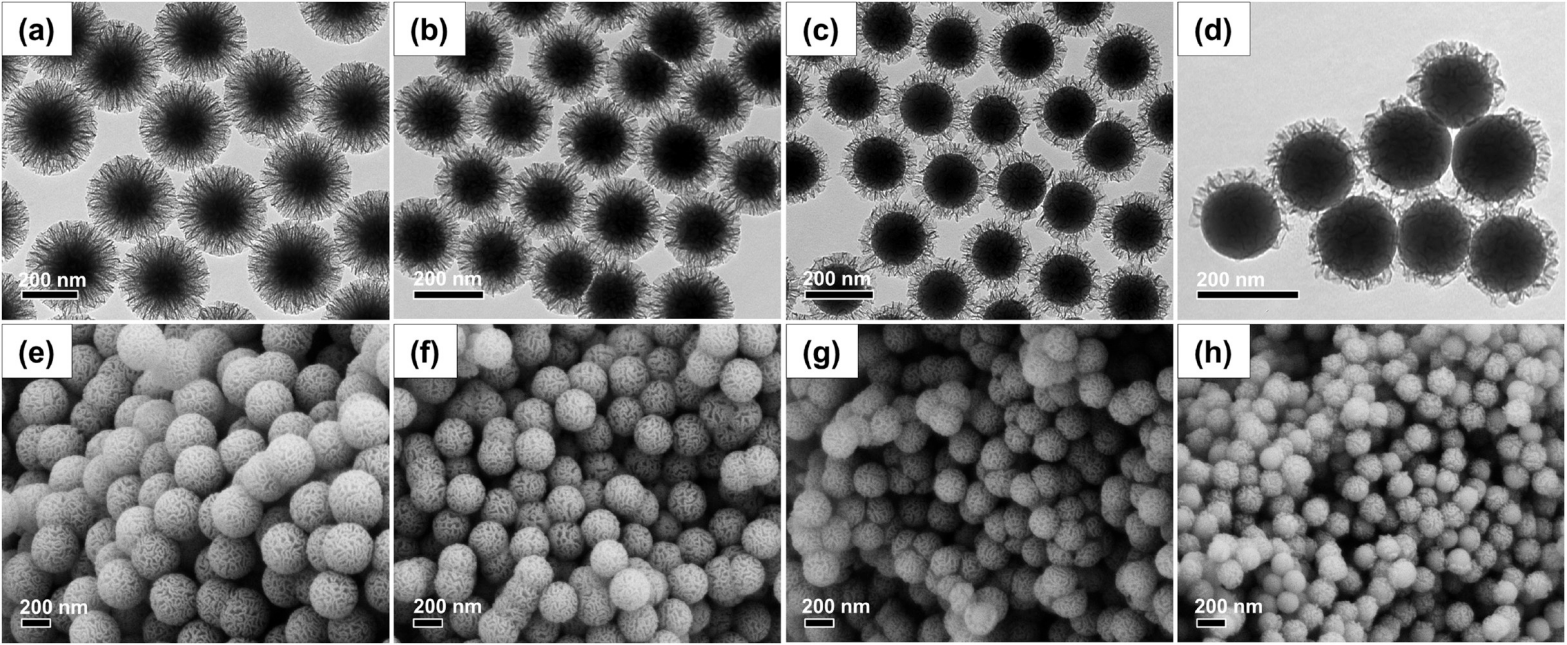
TEM images of (a) DMSNs@Ssilica-18, (b) DMSNs@Ssilica-7, (c) DMSNs@Ssilica-4, and (d) DMSNs@Ssilica-2. (e–h) corresponding SEM images.

**Figure 3:**
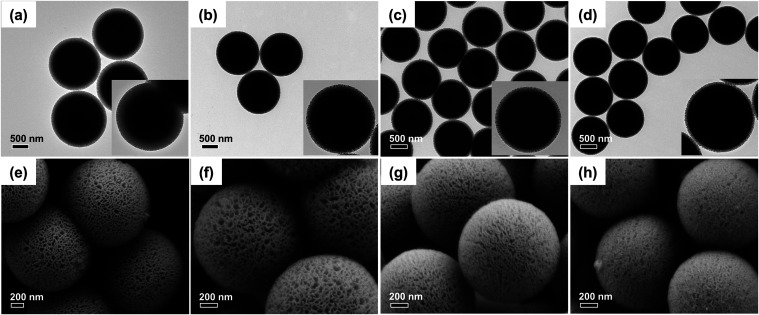
TEM images of (a) DMSNs@Bsilica-7, (b) DMSNs@Bsilica-4, (c) DMSNs@Bsilica-2, and (d) DMSNs@Ssilica-1. (e–h) corresponding SEM images.

The same trend was observed for DMSNs@Bsilica (Figure 3a–d), where particle size decreased with decreasing R. The average particle sizes from TEM were 1.46 ± 0.04, 1.34 ± 0.02, 1.29 ± 0.06, and 1.18 ± 0.03 μm for DMSNs@Bsilica-7, -4, -2, and -1, respectively. Because the 1 μm silica cores possess a lower specific surface area, a higher mass of core particles was required compared to smaller cores. At *R* = 18 (high core-TEOS mass ratio), both heterogeneous growth on the cores and homogeneous nucleation in solution occurred, leading to the coexistence of DMSNs@Bsilica and discrete DMSNs particles. A summary of the material properties before and after coating is shown in Table 1.

**Table 1:** Summary of the Material Properties and Core Particle-TEOS Mass Ratios Used for Synthesis, and the Material Properties of the Resulting Core–Shell Structures Synthesized Using the Anion-Assisted Method^a^

core material properties			after coating			
particle	mass (g)	particle:TEOS mass ratio	sample name	TEM shell thickness (nm)	BET SSA (m^2^ g^–1^)	BET pore volume (cm^3^ g^–1^)
small silica (172 ± 7 nm, 4 m^2^ g^–1^)	0.2	1:18	DMSNs@Ssilica-18	87 ± 7	329 ± 10	0.83 ± 0.15
	0.5	1:7	DMSNs@Ssilica-7	41 ± 6	208 ± 27	0.80 ± 0.03
	1.0	1:4	DMSNs@Ssilica-4	30 ± 6	145 ± 22	0.44 ± 0.07
	1.5	1:2	DMSNs@Ssilica-2	19 ± 5*	68 ± 2	0.22 ± 0.04
big silica (1.16 ± 0.30 μm, 3 m^2^ g^–1^)	0.5	1:7	DMSNs@Bsilica-7	151 ± 23	145 ± 18	0.31 ± 0.02
	1.0	1:4	DMSNs@Bsilica-4	91 ± 17	74 ± 9	0.19 ± 0.04
	1.5	1:2	DMSNs@Bsilica-2	65 ± 32	56 ± 2	0.20 ± 0.01
	3.0	1:1	DMSNs@Bsilica-1	11 ± 17	25 ± 2	0.09 ± 0.01

a The * indicates uneven coating.

These observations are consistent with previous studies, which have shown that heterogeneous nucleation of silica on core particles can be explained by combining LaMer nucleation theory with the influence of surface area.^39^ During TEOS hydrolysis, silica monomers accumulate in solution; when their concentration is moderate, silica preferentially deposits on the surface of the core particles (heterogeneous nucleation). However, if the monomer concentration becomes too high, then new silica particles form in the bulk solution (homogeneous nucleation). By increasing the amount of core particles while keeping the amount of TEOS constant, the total available surface area increases, allowing monomers to be consumed through surface growth and suppressing the formation of free SiO_2_ nanoparticles. Importantly, larger silica cores provide less total surface area per unit mass than smaller ones, so the optimal coating conditions differ for large and small particles and should be adjusted accordingly to ensure controlled shell formation.

Furthermore, the formation of dendritic silica shells in micelle-templated sol–gel systems depends strongly on surfactant micelle concentration, TEOS concentration, and available surface area for heterogeneous nucleation.^40–42^ Under constant precursor conditions, increasing the amount of core particles increases the total surface area, thereby reducing the effective TEOS (decreasing R) and the micelle concentration per unit surface. This results in smaller shells and, at a sufficiently high core amount, insufficient micelle coverage to template continuous radial growth.

Nitrogen adsorption–desorption analysis was performed to evaluate the surface properties of the core–shell materials. The BET surface areas were 329 ± 10, 208 ± 27, 145 ± 22, and 68 ± 2 m^2^ g^–1^ for DMSNs@Ssilica-18, -7, -4, and -2, respectively, while the bare silica cores exhibited a surface area of only 4 m^2^ g^–1^. The BET surface area for DMSNs@Bsilica is also listed in Table 1. This substantial increase confirms that the DMSNs shell is the dominant contributor to the overall surface area of the core–shell materials. The gradual decrease in BET surface area with decreasing core particle-TEOS ratio corresponds to a systematic reduction in shell thickness, demonstrating that the thickness of the dendritic silica layer can be tuned by varying the amount of core particles introduced during synthesis. Under constant TEOS and surfactant concentrations, increasing the core particle concentrations used for synthesis increases the total available surface area, thereby lowering the effective precursor-to-surface ratio. This provides a straightforward strategy to control shell thickness and tailor the material’s overall surface area without altering the reaction composition.

All samples exhibit type IV adsorption–desorption isotherms characteristic of mesoporous materials, with a pronounced capillary condensation step at high relative pressure *P*/*P*_0_ ≈ 0.75–0.95 (Figures 4a and 5a), confirming the presence of mesopores consistent with the wrinkled lamellar architecture of DMSNs. The Barrett–Joyner–Halenda (BJH) pore size distribution curves of DMSNs@Ssilica-R, calculated from the desorption branch, are presented in Figure 4b.

**Figure 4:**
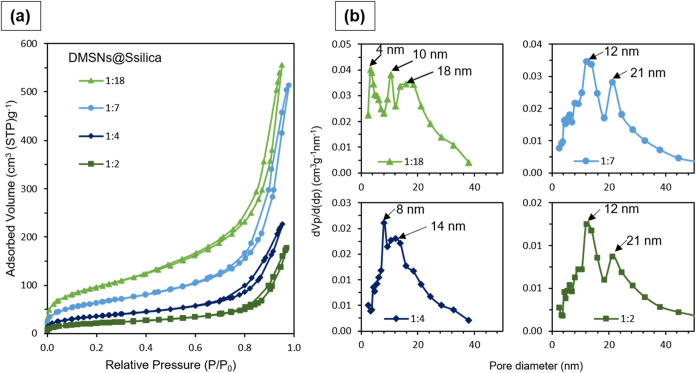
(a) Nitrogen adsorption–desorption isotherm of DMSNs@Ssilica-18, -7, -4, and -2. (b) Corresponding BJH pore size distribution obtained at different volume percentages.

**Figure 5:**
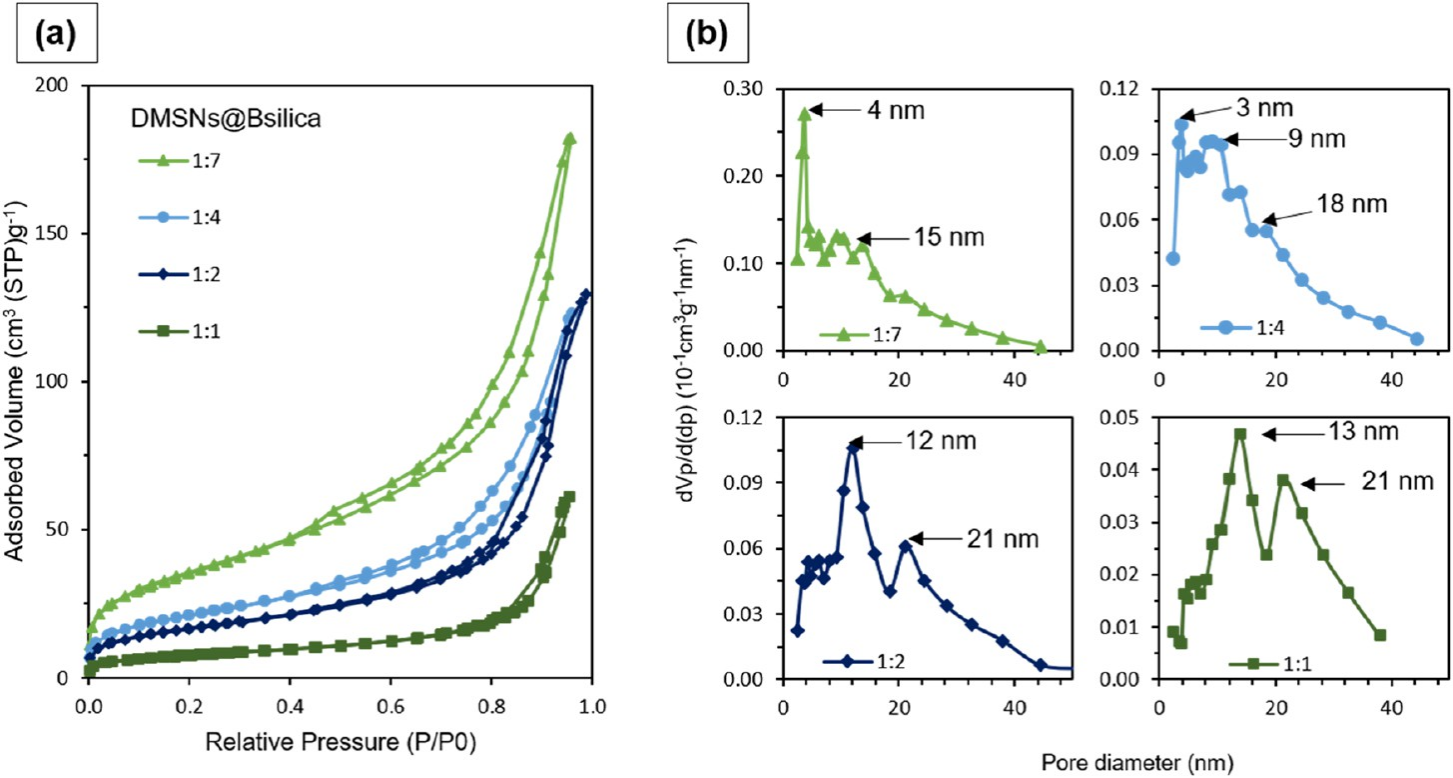
(a) Nitrogen adsorption–desorption isotherm of DMSNs@Bsilica-7, -4, -2, and -1. (b) Corresponding BJH pore size distribution obtained at different volume percentages.

The BJH method assumes capillary condensation/evaporation in cylindrical pores, which does not fully capture the dendritic architecture, but the analysis provides a reliable comparative assessment of the hierarchical porosity. For DMSNs@Silica-18 (Figure 4b), three distinct peaks appear at 4, 10, and 18 nm, with the most intense peak at 4 nm, indicating a significant contribution from small mesopores. As the ratio of core particle to TEOS decreases (DMSNs@Ssilica-7, -4, and -2), the 4 nm peak disappears, and the first observable peaks shift to larger pore sizes (8–14 nm), while the second peaks appear at 21 nm for DMSNs@Ssilica-7 and DMSNs@Ssilica-2. For larger cores, this systematic shift is observed in the last two samples (Figure 5B). The 4 nm peak represents the major peak for DMSNs@Bsilica-7 and DMSNs@Bsilica-4 but also disappears with a lower core particle-TEOS ratio. The first observable peaks shift to larger pore sizes (12–15 nm) for DMSNs@Bsilica-2, and -1, while the second peaks appear at 21 nm, similar to DMSNs@Ssilica.This systematic shift toward larger pore diameters with increasing R reflects the reduction of the smallest pores and demonstrates that the pore size distribution of the DMSN shell can be tuned by adjusting the ratio of core particle to TEOS used in the synthesis.

Finally, the pore volume of the core–shell materials was estimated from the BJH analysis (Table 1). Although DMSNs@Ssilica-18 exhibits a significantly higher surface area than DMSNs@Ssilica-7, their pore volumes remain relatively similar, measuring 0.83 ± 0.15 and 0.80 ± 0.03 cm^3^ g^–1^, respectively. In contrast, DMSNs@Ssilica-4 and DMSNs@Ssilica-2 show significantly lower pore volumes compared to the former two samples. When these results are considered alongside the pore size distribution data, it becomes evident that a broader or larger pore size distribution contributes more significantly to increased pore volume than surface area alone. A similar trend is observed for the larger core–shell materials: DMSNs@Bsilica-4 and DMSNs@Bsilica-2 exhibit pore volumes of approximately 0.20 ± 0.01 cm^3^ g^–1^, comparable to that of DMSNs@Bsilica-7, despite having substantially lower surface areas.

The nitrogen sorption data from DMSNs@Ssilica-R provide important insight into which structural parameter governs pore evolution in this system, specifically whether changes in pore size arise from variations in interwrinkle distance or from nondendritic silica-coated micelles (Figure 5). All samples exhibit type IV isotherms with capillary condensation occurring consistently at high relative pressures (*P*/*P*_0_ ≈ 0.75–0.95). Importantly, this condensation region remains essentially unchanged as the core amount increases. According to the Kelvin equation, capillary condensation pressure is directly related to pore diameter. Therefore, a significant alteration in interwrinkle distance would produce a systematic shift in condensation pressure. The absence of such a shift strongly indicates that the primary interwrinkle pore diameter is preserved across the series, as is also evident in the SEM images (Figures 2 and 3e–h). If interwrinkle distance were the dominant structural variable controlling pore evolution, increasing core amount would be expected to alter fiber packing density and therefore modify the large pore size. However, the invariance of the condensation step suggests that the radial lamellar structure remains the same regardless of shell thickness. This observation argues against interwrinkle expansion as the main driver of the observed changes in surface area and pore distribution.

In contrast, the BJH pore size distributions reveal substantial modifications in fine mesoporosity (Figure 5b). At low core mass (DMSNs@Ssilica-18), three distinct pore peaks are observed (∼4, 10, and 18 nm), with the strongest contribution at ∼4 nm. As the core amount increases, the ∼4 nm peak disappears and the distribution shifts toward larger radially oriented mesopores (8–14 nm and ∼21 nm), accompanied by a pronounced decrease in BET surface area (from 329 to 68 m^2^ g^–1^). Because the primary condensation pressure remains constant, these changes cannot originate from enlargement of the interwrinkle voids. Instead, they reflect a reduction in the number of smaller micelle-derived mesopores.

This trend is consistent with a dynamic pore-filling mechanism operating within an otherwise stable lamellar framework. In anion-assisted systems, micelles and silicate species coexist throughout the condensation process. After the initial lamellar structure forms, residual composite micelles can infiltrate the large voids and deposit along the lamella surfaces through a continued silanol condensation. Upon calcination, the removal of these embedded micelles generates smaller mesopores within the walls of the interwrinkle channels, increasing the surface area and hierarchical complexity.

The amount of core particles used in the synthesis introduces an additional kinetic constraint. Increasing the mass of silica cores increases the total available surface area while precursor concentrations remain constant. Consequently, the effective TEOS-to-surface and micelle-to-surface ratios decrease. Under these conditions, silicate species are preferentially consumed in coating growth rather than secondary micelle deposition inside the radially oriented interwrinkle mesopores. Reduced micelle infiltration results in the disappearance of the ∼4 nm pores and a decline in surface area, while the large interwrinkle pores remain unchanged. In contrast, at higher TEOS-to-core ratios, excess TEOS is available for condensation around micelles that infiltrate the growing lamellar DFNS structure, giving rise to the additional ∼4 nm mesopores observed in DMSNs@Ssilica-18 and DMSNs@Bsilica-7. Therefore, the observed structural changes across DMSNs@Ssilica-R and DMSNs@Bsilica-R are better explained by the micelle-mediated pore-filling mechanism rather than changes in interwrinkle spacing. The interwrinkle distance contributes to large pores and high pore volume, important for enhanced transport channels, while micelle infiltration controls small mesoporosity and large surface area.

This behavior highlights a key advantage of the anion-assisted approach: hierarchical porosity can be tuned without altering fundamental synthesis parameters such as surfactant type, anion species, pH, temperature, or reaction time. This tunability is particularly significant because it allows optimization of transport pathways and accessible surface area independently. Large dendritic or wrinkled lamellar channels facilitate mass transfer, whereas smaller mesopores enhance surface area and adsorption capacity. By controlling the core-TEOS ratio alone, the balance between diffusion efficiency and surface functionality can be systematically tailored.

To understand the role of pore architecture and shell thickness on mass transport and loading behavior, silver nanoparticles (Ag NPs, ∼4 nm, PEI surface-capped) were electrostatically immobilized within DMSNs@Ssilica-18 and DMSNs@Ssilica-7. These two systems differ primarily in their pore distribution and shell dimensions: DMSNs@Ssilica-18 contains both large radially oriented interwrinkle mesopores and smaller secondary mesopores (∼4 nm) with a thicker shell, whereas DMSNs@Ssilica-7 consists predominantly of interwrinkle channels with minimal pore constrictions and a comparatively thinner shell.

Qualitative EDS elemental mapping reveals substantially higher Ag intensity in DMSNs@Ssilica-7 relative to DMSNs@Ssilica-18 (Figure 6a–d), indicating enhanced nanoparticle uptake. Because the Ag nanoparticles are comparable in size to the smallest mesopores in DMSNs@Ssilica-18, partial steric restriction and pore blocking are expected. The presence of narrow mesopores likely introduces local diffusion barriers, increasing tortuosity and reducing effective pore accessibility.^29,43,44^ Under diffusion-controlled conditions, nanoparticle transport within porous solids can be described by Fick’s law, where the flux is proportional to the concentration gradient across the shell. In porous materials, the effective diffusivity of nanoparticles is lower than in a bulk solution due to the combined effects of porosity, tortuosity, and steric hindrance.^29,44^ When the functional particle size approaches the pore diameter, steric constraints reduce the fraction of accessible pore space, lowering the effective diffusivity.^43^ Narrow pore constrictions and curved pathways further increase tortuosity, slowing diffusion through the shell.

**Figure 6:**
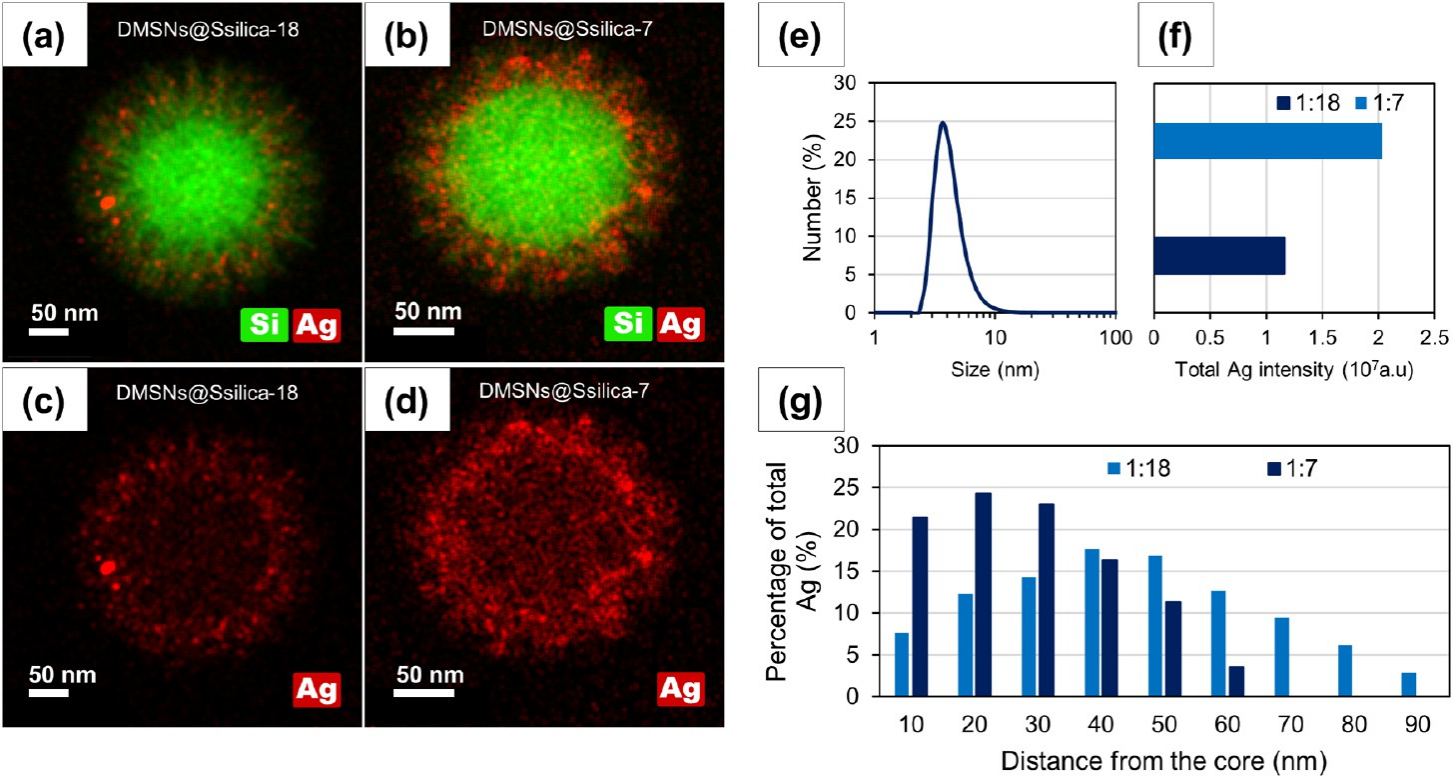
STEM-EDS elemental mapping of DMSNs@Ssilica-18-Ag (left panel) and DMSNs@Ssilica-7-Ag (middle panel), showing (a, b) silver (red) distribution overlaid on silica (green) and (c, d) corresponding silver maps. (e) Size distribution of Ag nanoparticles, (f) total Ag intensity within the particle, and (g) percentage Ag distribution as a function of distance from the core.

To semiquantitatively assess radial distribution, pixel intensity profiles were extracted from the EDS maps using Python-based image analysis and correlated with radial distance from the core. The integrated Ag intensity in DMSNs@Silica-7 is approximately 2-fold higher than that in DMSNs@Silica-18 (Figure 6f), confirming significantly improved loading capacity despite comparable overall pore volumes.

The radial intensity profiles reveal distinct transport behaviors (Figure 6g). In DMSNs@Ssilica-18, the Ag distribution follows a near-Gaussian profile with the maximum intensity located ∼40 nm from the surface of the 2d projection and ∼50% of the cumulative intensity residing in the inner shell region. This distribution suggests progressive diffusion with increasing resistance toward the outer shell, consistent with pore constrictions and partial steric hindrance.^29,43^ The presence of small mesopores likely increases internal surface area but does not proportionally enhance accessible volume for nanoparticles of this size, thereby decoupling surface area from effective loading capacity. In contrast, DMSNs@Ssilica-7 exhibits a non-Gaussian distribution, with ∼70% of the Ag intensity concentrated within ∼20 nm of the core–shell interface. As discussed above, the absence of smaller mesopores likely reduces diffusional bottlenecks, lowers tortuosity, and enhances effective diffusivity within the interwrinkle channels. As a result, nanoparticles penetrate more efficiently and accumulate preferentially in regions governed primarily by electrostatic interactions rather than steric exclusion.

These findings demonstrate that for nanoparticle-sized guests, pore connectivity and minimum pore diameter dominate over the total surface area in determining loading efficiency. Architectures containing only large, radially oriented interwrinkle mesopores provide reduced transport resistance and greater effective pore accessibility, validating the design principle that eliminating pore constrictions within the radial channels enhances diffusion-controlled loading processes in core–shell DMSN materials.

To further validate the transport-based interpretation established from the Ag nanoparticle study, lysozyme was electrostatically immobilized into all core–shell samples and compared with discrete DMSNs. Lysozyme, with a hydrodynamic diameter of ∼4 nm and a positive surface charge at neutral pH,^45^ is particularly sensitive to pore size constraints^26^ and therefore serves as an ideal probe to assess steric hindrance and diffusion limitations within the lamellar shell, especially since the smallest pore size in the DMSNs materials is comparable to its size. As expected, discrete DMSNs exhibit a higher total lysozyme loading (538.6 mg g^–1^) than the DMSNs@Ssilica-R and DMSNs@Bsilica-R core–shell materials (Figure 7a,c). This difference arises because the core–shell structures contain a nonporous core that contributes to the total particle mass but does not provide additional adsorption sites, thereby reducing the overall loading capacity when expressed per gram of material. Notably, this loading capacity is significantly higher than previously reported values for DMSNs synthesized using sodium trifluoroacetate as the counterion, which showed a lysozyme loading of 152.4 mg g^–1^.^26^ The lower loading in that case can be attributed to the substantially smaller specific surface area (349 m^2^ g^–1^) compared to that of the DMSNs reported here.

**Figure 7:**
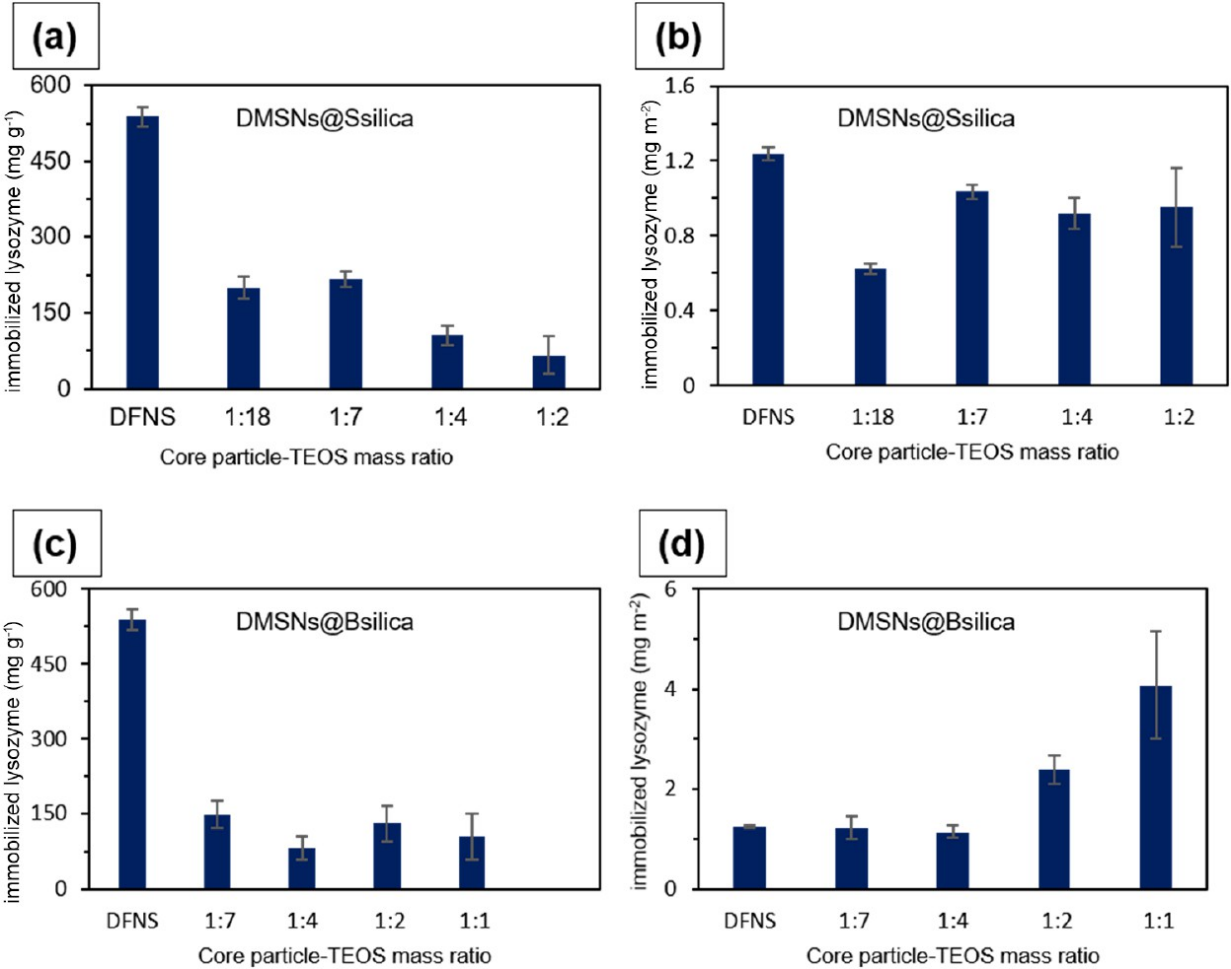
Enzyme loading capacity of DMSNs@Ssilica (top) and DMSNs@Bsilica (bottom): (a, c) normalized to mass; (b, d) normalized to surface area.

Trends within the different core particle-TEOS ratios reveal behavior consistent with the previously established diffusion-controlled loading mechanism observed for Ag nanoparticles. DMSNs@Ssilica-18 and DMSNs@Ssilica-7, which possess comparable pore volumes but differ in pore size range and shell thickness, both immobilize approximately 200 mg g^–1^ of lysozyme. In contrast, DMSNs@Ssilica-4 and DMSNs@Ssilica-2, with reduced pore volume and surface area, exhibit lower adsorption capacities (Figure 7a). A similar pattern is observed in the larger core–shell structures: DMSNs@Bsilica-7 and DMSNs@Bsilica-4 show comparable lysozyme loadings (∼140 mg g^–1^) despite differences in specific surface area and pore size distribution (Figure 7c), but similar pore volumes. Importantly, these trends mirror the Ag nanoparticle results, where loading was influenced by effective diffusivity rather than surface area alone. Because the size of lysozyme (∼4 nm) closely matches the smallest mesopores present in some samples, the introduction of sub-5 nm pores imposes steric constraints that reduce effective diffusivity within the shell. Thus, even when surface area is high, the accessible pore volume for enzyme molecules is diminished by steric hindrance and increased tortuosity, analogous to the diffusion limitations observed for Ag nanoparticles in DMSNs@Ssilica-18.

When adsorption capacity is normalized to immobilized enzyme per unit surface area (mg m^–2^), this transport limitation becomes even more evident. For DMSNs@Ssilica-18 and DMSNs@Ssilica-7, the latter exhibits significantly greater surface utilization, where adsorption capacity was 0.6 and 1.0 mg m^–2^, respectively (Figure 7b), indicating that the absence of small mesopores improves effective accessibility of the internal surface. Among samples containing exclusively large radially oriented interwrinkle mesopores (DMSNs@Silica-7, -4, -2), surface-normalized loading remains relatively consistent at 1.0 mg m^–2^, suggesting that once steric bottlenecks are eliminated, adsorption becomes primarily governed by available surface area rather than diffusion resistance.

The same trend is observed for the larger core–shell materials. DMSNs@Bsilica-7 and DMSNs@Bsilica-4, which contain small mesopores, display similar surface-area-normalized loading efficiencies (∼1.2 mg m^–2^), whereas DMSNs@Bsilica-2 and DMSNs@Bsilica-1, possessing exclusively large radially oriented interwrinkle mesopores, exhibit improved immobilization efficiency of 2.3 and 4.1 mg m^–2^ (Figure 7d). Notably, the DMSNs@Bsilica-R series generally achieves higher enzyme immobilization per unit surface area than the Ssilica analogues. This can be attributed to a high degree of agglomeration of smaller particles compared to large particles, leading to reduced exposed surface for enzyme interaction.^46^

In previous studies using DMSNs with similar surface areas but different particle sizes (79 and 160 nm), the enhanced lysozyme loading observed for smaller particles containing only large pores was attributed primarily to particle size effects.^26^ However, the influence of small mesopores present in the larger particles was not fully considered. Although the larger particles contained pores up to 22 nm, the presence of smaller pores (∼2.7 nm) likely restricted lysozyme diffusion into the larger cavities, thereby reducing effective loading. Importantly, despite the pore diameters being substantially larger than the lysozyme molecule, sustained enzyme release over 48 h with enhanced antimicrobial activity against *E. coli* was observed.^26^ This indicated that the immobilized enzyme remained stable within the 22 nm pores without significant leaching; otherwise, a burst release would have occurred, given the size mismatch between the enzyme and the pore voids.^47^

Taken together with the Ag nanoparticle distribution analysis, these results confirm that loading behavior in core–shell DMSNs materials is governed by diffusion-controlled transport, where effective diffusivity is dictated by minimum pore diameter, tortuosity, and shell thickness. Surface area alone does not determine loading efficiency; rather, the fraction of surface that is kinetically accessible under unhindered diffusion controls immobilization performance. Eliminating subcritical mesopores and optimizing shell thickness, therefore, enhances accessible pore volume and maximizes loading efficiency for both nanoparticle and enzymatic functional materials.

## Conclusion

In summary, we report a simple and scalable anion-assisted strategy using sodium salicylate to tune the pore size and shell thickness in DMSN silica core–shell particles. By varying only the core-to-TEOS ratio, hierarchical porosity was adjusted in both nano- and microscale systems without modifying the surfactant composition or reaction conditions. Increasing the core content affects the TEOS availability during shell formation. TEOS first hydrolyzes to silanol groups and then condenses to form the Si–O–Si framework of the lamellar shell. In the initial stage, much of the TEOS is consumed, building the primary lamellar structure around the cores, leaving less precursor for secondary micelle filling that generates small mesopores. As a result, a higher core content results in limited secondary pore formation while preserving the primary wrinkled lamellar architecture. This behavior is consistent with a micelle-filling mechanism, highlighting the role of precursor availability in controlling hierarchical pore development.

Transport and adsorption studies with silver nanoparticles and lysozyme demonstrate that diffusion limitations rather than total surface area alone dictate functional performance. Importantly, we show that pore accessibility within the large interwrinkle channels is strongly influenced by the presence of smaller mesopores embedded within them. These small pores act as steric and diffusion bottlenecks, reducing effective surface utilization, whereas materials containing exclusively large radially oriented interwrinkle mesopores exhibit improved accessibility and higher loading efficiency.

Overall, the core-TEOS ratio serves as a single-parameter design handle to balance the pore accessibility and adsorption capacity. These findings highlight that careful control of pore architecture is essential when designing DMSN materials for the loading of larger molecules. Given the complex, radially oriented lamellar pore structure of DMSNs, absolute pore sizes should be interpreted with caution; however, comparative trends between samples remain reliable and support the mechanistic conclusions presented here. Materials with exclusively large radially oriented interwrinkle mesopores are ideal for loading and releasing larger molecules, whereas the complex mesopore structure enables selective adsorption, controlled release, or size-dependent catalysis, where precise control of accessibility and surface area is critical. Importantly, the cores impart intrinsic properties to the core–shell materials, providing additional functionalities that complement the porous shell. For example, large core–shell DMSNs loaded with enzymes can be employed in bioprocess systems such as flow-through or depth-filter setups for efficient cell lysis or catalytic transformations. Beyond biocatalysis, the combination of tunable porosity and core functionality also opens opportunities in sensing, hybrid nanomaterials, and other chemical or biological applications, where both structural control and inherent core properties are essential.
